# Corrigendum: The cGAS-STING pathway affects vertebral bone but does not promote intervertebral disc cell senescence or degeneration

**DOI:** 10.3389/fimmu.2023.1201655

**Published:** 2023-04-26

**Authors:** Olivia K. Ottone, C. James Kim, John A. Collins, Makarand V. Risbud

**Affiliations:** ^1^ Department of Orthopaedic Surgery, Sidney Kimmel Medical College, Thomas Jefferson University, Philadelphia, PA, United States; ^2^ Graduate Program in Cell Biology and Regenerative Medicine, Jefferson College of Life Sciences, Thomas Jefferson University, Philadelphia, PA, United States

**Keywords:** Intervertebral disc, cGAS-STING, SASP, aging, nucleus pulposus, inflammation, vertebrae, senescence

In the published article, an author name was incorrectly written as “Cheeho Kim”. The correct spelling is “C. James Kim”.

The authors apologize for this error and state that this does not change the scientific conclusions of the article in any way. The original article has been updated.

