# Supplementary material for: The cGAS-STING Pathway Affects Vertebral Bone but Does Not Promote Intervertebral Disc Cell Senescence or Degeneration
Source: Front Immunol. 2022 Jun 13;13:882407. doi: 10.3389/fimmu.2022.882407 (PMC9235924; doi:10.3389/fimmu.2022.882407)
Supplement: Supplementary file 1 [file DataSheet_1.pdf]

Suppl. Figure 1

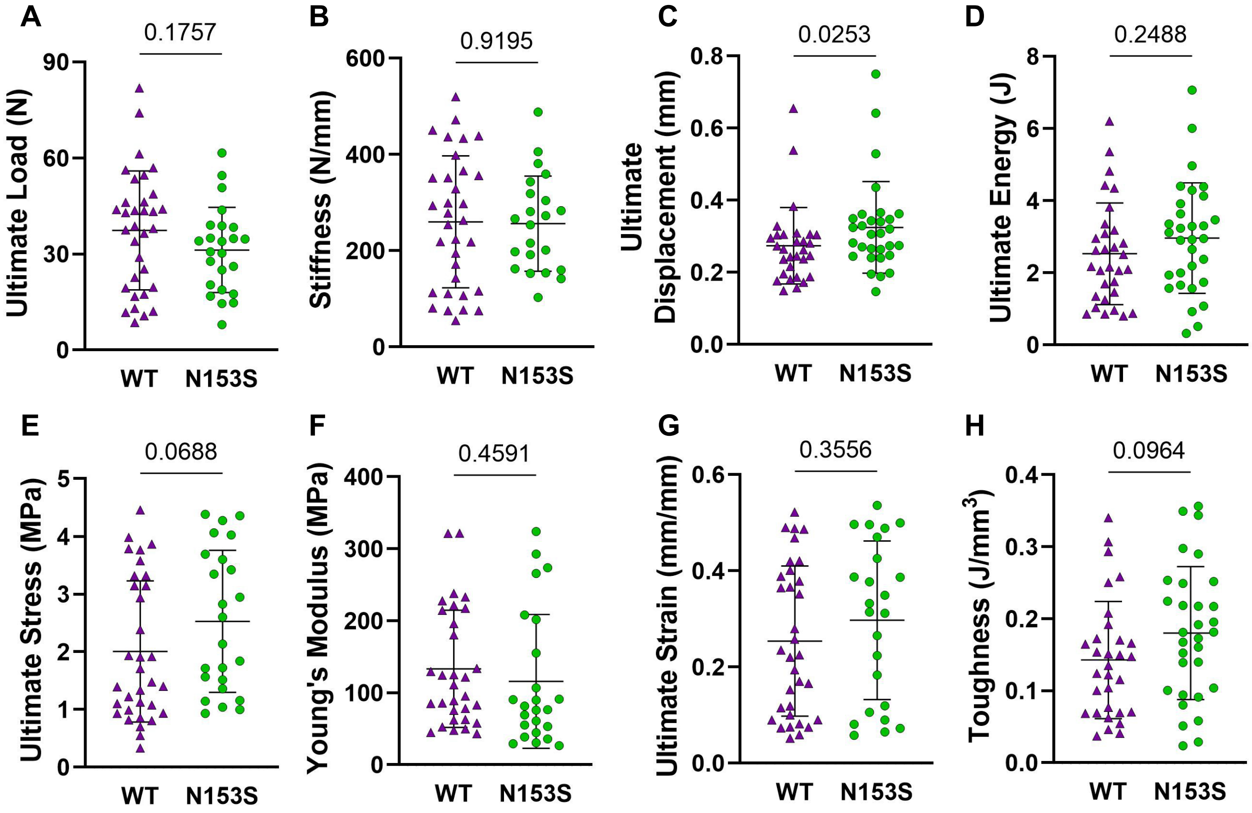

Supplementary Figure 1. Changes in N153S vertebral architecture do not lead to functional changes. WT and N153S caudal vertebral bone structural properties of (A) ultimate load, (B), stiffness, (c) ultimate displacement, and (D) ultimate energy and their corresponding material properties of (E) ultimate stress, (F) Young's Modulus, (G) ultimate strain, and (H) toughness, as determined by compression testing. (n=2-3 caudal vertebrae/animal, 12 animals/genotype, 27-32 vertebrae/genotype). Data are represented as the mean ± SD. Significance was determined using an unpaired t-test or Mann-Whitney test, as appropriate.

Suppl. Figure 2

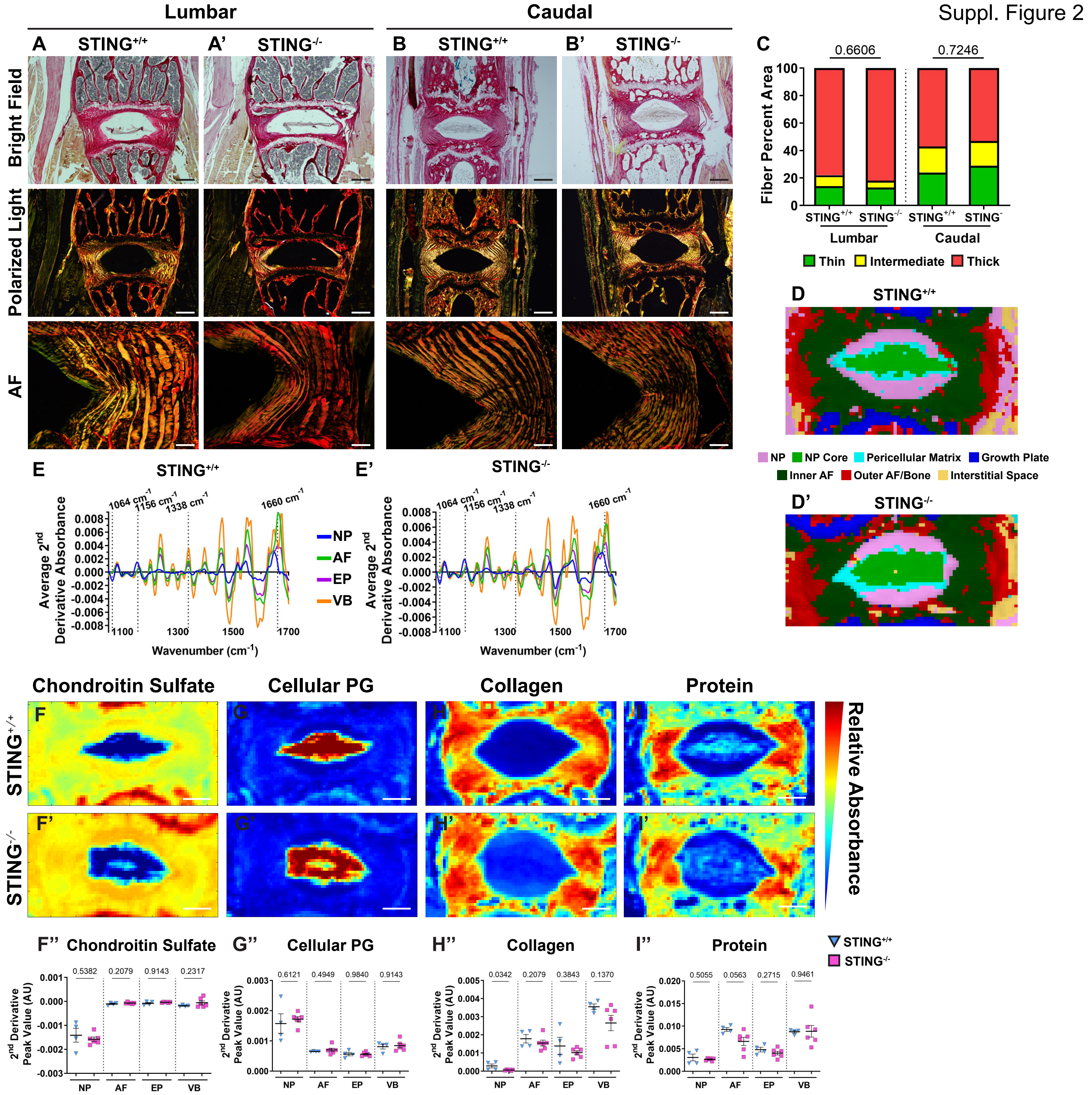

Supplementary Figure 2. STING deletion does not alter collagen structure or chemical composition in the aging mouse disc.

(A-B') Picrosirius Red staining of 16-18-month-old (A, A') lumbar and (B, B') caudal discs showing collagen organization of the AF in the bright field (top row) and collagen fiber distribution under polarized light (middle and bottom rows) (scale bar= 100 µm). (C) Quantification of fiber thickness distribution (n=5 lumbar discs, 4 caudal discs/animal, 6 animals/genotype, 30 lumbar discs/genotype, 24 lumbar discs/genotype). (D, D') Average second derivative spectra, inverted for positive visualization, of the NP, AF, EP, and vertebrae (VB) of (H, H') of 16-18-month-old $STING^{+/+}$ and $STING^{-/-}$ mice (n=1 lumbar disc/animal, 4 animals/genotype, 4 total discs/genotype). (E, E') Spectral cluster analysis images (Scale bar = 200 µm). (F-H') Chemical maps and (F"-H") quantification of mean second derivative peaks for (F-F") chondroitin sulfate (1064 cm^{-1}), (G-G") collagen (1338 cm^{-1}), and (H-H") total protein (1660 cm^{-1}) content. Significance between fiber distribution was determined using a χ^2 test. AU: arbitrary units. Quantitative measurements represent mean ± SD. Significance of chemical components was determined using an unpaired t-test or Mann-Whitney test, if data were not normally distributed.

Suppl. Figure 3

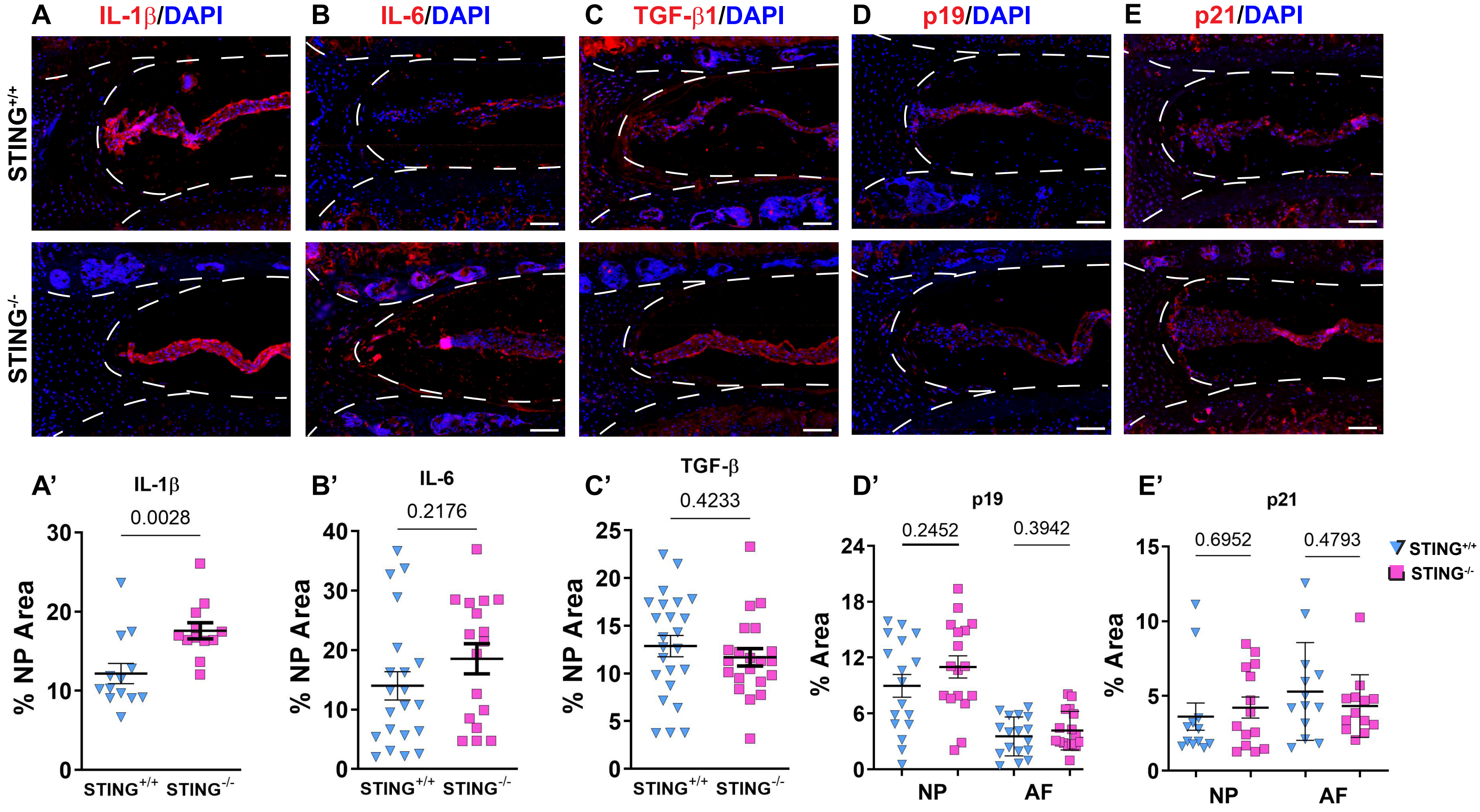

Supplementary Figure 3. SASP induction is not delayed in STING$^{-/-}$ mice.
(A-D') Quantitative immunohistological staining of 16-18-month-old STING$^{+/+}$ and STING$^{-/-}$ mice lumbar discs for: (A, A') IL-1β, (B, B') IL-6, (C, C') TGF-β), (D, D') p19, and (E, E') p21. Images were taken at 10x (scale bar= 100 µm). (n=2-4 discs/animal; 6 animals/genotype, 12-24 total discs/genotype) Dotted lines demarcate different tissue compartments within the disc. Quantitative data represents the mean ± SD. Significance was determined using unpaired t-test or Mann-Whitney test, as appropriate.
